# Ceramic Bone Graft Substitutes do not reduce donor-site morbidity in ACL reconstruction surgeries: a pilot study

**DOI:** 10.1051/sicotj/2019013

**Published:** 2019-05-14

**Authors:** Naresh Dhanakodi, Jai Thilak, Jacob Varghese, Krishnankutty Venugopal Menon, Harikrishna Varma, Sujit Kumar Tripathy

**Affiliations:** 1 Department of Orthopaedics, Meenakshi Mission Hospital Madurai India; 2 Department of Orthopaedics, Amrita Institute of Medical Sciences Kochi India; 3 Department of Orthopaedics, Lakeshore Hospital and Research Centre Kochi India; 4 Department of Orthopaedics, Sparsh Hospital Bangalore India; 5 Bioceramic lLaboratory, Sree Chitra Tirunal Institute for Medical Sciences Trivandrum India; 6 Department of Orthopaedics, AIIMS Bhubaneswar India

**Keywords:** Bone-patellar-tendon-bone graft, Anterior cruciate ligament, Arthroscopy, Hydroxyapatite–Bioglass ceramic, Bone graft substitute

## Abstract

*Introduction:* Anterior knee pain is a major problem following Bone-patellar-tendon-bone graft (BPTB) use in anterior cruciate ligament (ACL) reconstruction. We hypothesized that filling the donor defect sites with bone-graft substitute would reduce the anterior knee symptoms in ACL reconstruction surgeries.

*Material and Methods*: Patients operated for ACL-deficient knee between March 2012 and August 2013 using BPTB graft were divided into two treatment groups. The patellar and tibial donor-site bony defects were filled-up with Hydroxyapatite–Bioglass (HAP:BG) blocks in the study group (*n* = 15) and no filler was used in the control group (*n* = 16). At 2 years, the clinical improvement was assessed using International Knee Documentation Committee (IKDC) score and donor-site morbidity was assessed by questionnaires and specific tests related to anterior knee pain symptoms.

*Results*: Donor-site tenderness was present in 40% patients in the study group and 37.5% patients in the control group (*p* = 0.59). Pain upon kneeling was present in 33.3% patients in the study group and 37.5% patients in the control group (*p* = 0.55). Walking in kneeling position elicited pain in 40% patients in the study group and 43.8% in the control group (*p* = 0.56). The mean visual analogue score for knee pain was 3.0 in the study group and 3.13 in the control group, with no statistically significant difference (*p* = 0.68). Unlike control group, where a persistent bony depression defect was observed at donor sites, no such defects were observed in the study group.

*Conclusion:* Filling the defects of donor sites with HAP:BG blocks do not reduce the anterior knee symptoms in patients with ACL reconstruction using autogenous BPTB graft.

## Introduction

The BPTB graft has high initial tensile strength and stiffness, and shows excellent incorporation at both ends [1–4]. The major disadvantage of BPTB graft is about its donor-site morbidity. Anterior knee pain restricting the patient’s ability to kneel and walk while kneeling has been reported in 40%–60% patients and is one of the commonest morbidities of BPTB graft limiting its use [2, 5–8].

Hydroxyapatite (HAP) is a synthetic Calcium phosphate ceramic with close resemblance to natural bone mineral phase, structural strength, and biocompatibility but with limited degradation. Bioactive glasses belonging to Calcium phosphate silicate group have very good bonding properties with bone and soft tissues [9]. This is a biologically active compound and it encourages rapid new bone growth; as the surface integration is chemical in nature, tissue adhesion starts almost immediately and optimum interface strength is attained within weeks [9, 10]. This composite of HAP and bioactive glass ceramics presents beneficial properties of both the individual components [9]. Previous study has shown lesser donor-site morbidity after ceramic block application in the iliac crest bone graft donor site. Both ceramic materials have been used in spine and trauma surgeries and in Orthopaedic procedures [10–12].

**Figure 1:**
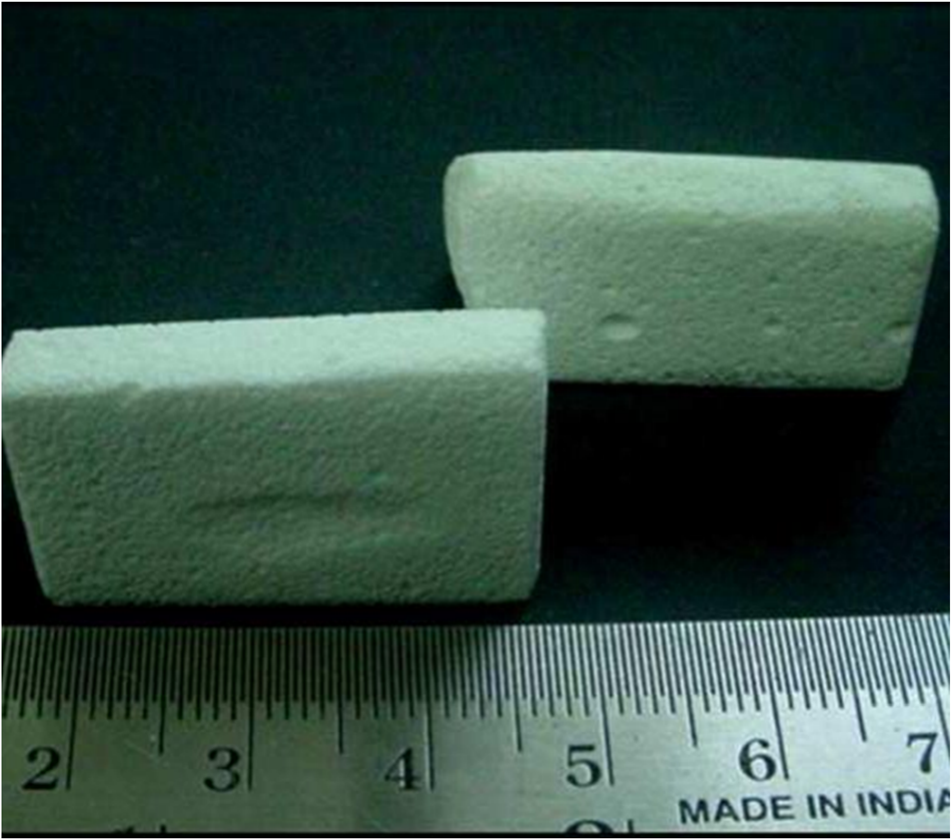
HAP:BG (BioOstin) ceramic block used in the study.

The objective of this study was to assess the effects of Hydroxyapatite:Bioglass (HAP:BG) ceramic filler used in the tibial and patellar donor sites of BPTB graft (Figure 1). It was hypothesized that filling donor sites with HAP:BG would reduce donor-site morbidity.

## Patients and methods

A prospective study was designed to evaluate the effect of Ceramic bone graft substitute on donor-site morbidity in patients undergoing ACL reconstruction surgeries using central third BPTB graft. Patients with ACL-deficient knee who presented to our clinic between March 2012 and August 2013 were evaluated clinically and radiologically for inclusion in this study (Table 1). Patients with multiple ligamentous injury or arthritic changes in the knee joint were excluded. Institutional Ethics and Research committee approval was obtained and patients were recruited into this study after getting their written informed consent.

**Table 1:** Patient demographics.

	Study group	Control group	*p* Value
No. of patients	15	16	
Mean age	32.4 years (20–43)	27.8 years (19–43)	0.026
Side	Left 6 (40%)	Left 7 (43.74%)	0.561
	Right 9 (60%)	Right 9 (56.25%)	(Chi-square test)
Time between injury and reconstruction in months	7 (1–37)	11(0.5–40)	0.199
Mean height (cm)	170.6	170.9	0.896
Mean weight (kg)	69.5	70.0	0.790

Arthroscopic assisted ACL reconstruction in these patients was performed through trans-tibial technique using central third BPTB graft and interference screw. All surgeries were performed by the senior surgeon. In every alternate patient, the donor sites were filled with HAP:BG blocks, and these patients formed the study group. Other patients, where the donor sites were not filled with any graft or graft substitute formed the control group. The demographics of the patients along with their clinical and radiological findings were entered into a predesigned computerized proforma.

### Graft harvesting technique

A traditional single vertical 3–4-cm incision was used for graft harvest. The central third of the tendon was dissected to create a 10–11-mm-wide graft. A narrow oscillating saw was used to harvest a tibial bone and the patellar bone block. The bone block was trimmed to form a trapezoidal block of 9–10-mm in diameter.

### Filling up the bony defect with ceramic bone substitute in the study group.

**Figure 2:**
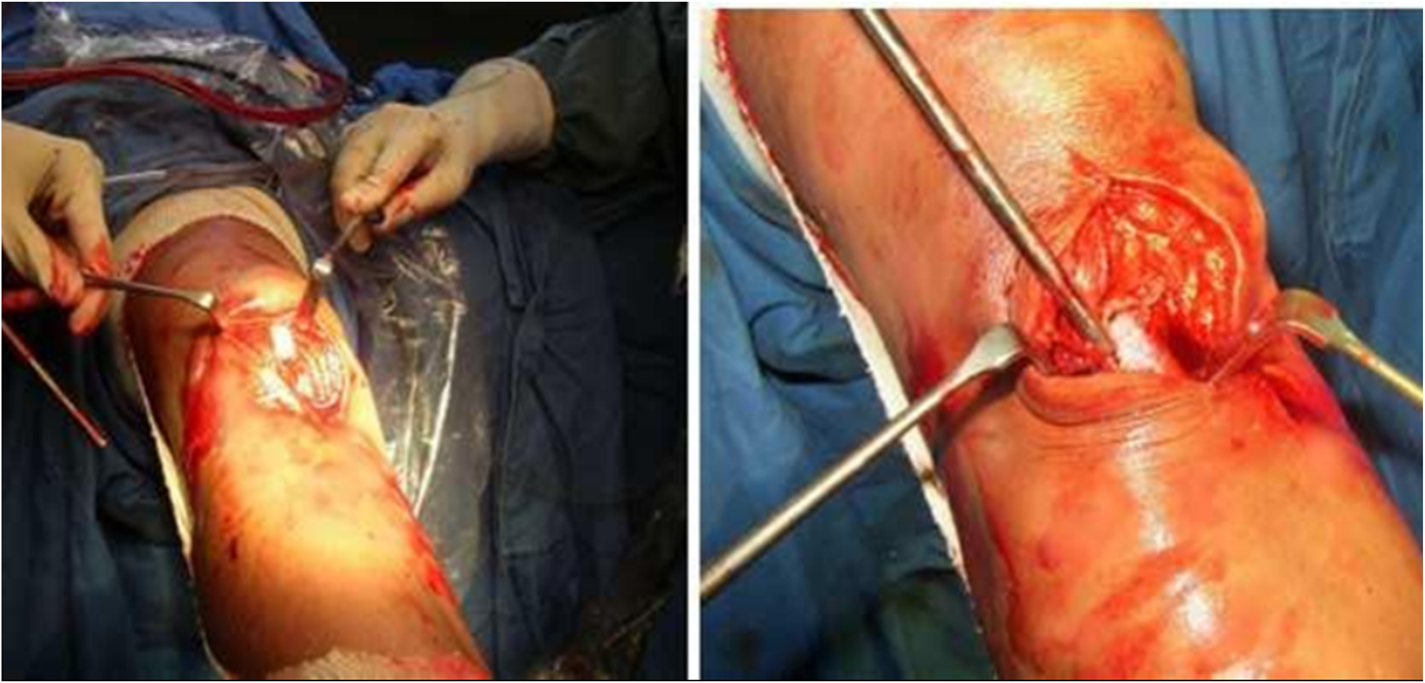
Photograph showing filling of the patellar and tibial bone defects with HAP:BG ceramic blocks.

The bony defects of the donor site were filled with HAP:BG (BioOstin^®^, Basic Health Care, India) blocks. The blocks are available in trapezoidal shape in two, three, and five centimetre lengths (Figure 1). The HAP:BG block of the preformed size was rasped and placed in the patella donor site. Similarly, the tibial donor defect was also packed with the block after appropriate sizing (Figure 2). The periosteum was sutured over the blocks and the paratenon sutured over the tendon.

### Wound closure in the control group

The paratenon was sutured loosely over the patellar tendon and the periosteum sutured over the donor site followed by the subcutaneous and skin closure.

### Postoperative rehabilitation and follow-up

The postoperative rehabilitation protocol remained similar in both groups. Motion control brace was used postoperatively. Closed chain exercises and knee ROM from 0–90 degree were started from the first post-op day and it was continued till two weeks. Gradual full knee ROM was encouraged between 2–4 weeks; hyperflexion of the knee was avoided during this period. Weight bearing as tolerated was allowed immediately after surgery. Sports activities were allowed after six months from the time of surgery. Both groups were followed up at six weeks, three months, six months, one year, and two years. The clinical and radiological outcomes were assessed by an Orthopaedic registrar (not involved in surgery and was unknown about the surgical procedure) and the results were tabulated. For clinical assessment, International Knee Documentation Committee (IKDC) score was used. Ligamentous stability was assessed by manual Lachman test and pivot shift test.

To evaluate donor-site morbidity in both groups, tenderness at the donor site was elicited and few questions were asked. (1) The patellar donor site was palpated for any tenderness and recorded as “tender” or “non-tender”. (2) Kneel pain was assessed by asking the patients to kneel on the bare knee and asked for the presence of pain in tibial donor site. (3) All patients were asked to perform “Knee walking test”. In this test, patients were asked to kneel on floor and take five steps forward on their knees without any protective clothing over their knee and questioned whether pain over the tibial donor site was present or not. (4) Patients were assessed as having retropatellar pain, if they had all of the following: (a) pain while resting with the knee flexed at more than 90°, (b) pain during or after the end of activity, (c) pain while walking the stairs up and down. (5) Visual analogue scale was used to evaluate subjective assessment of pain. Patients were asked to rate their pain from a scale of 0–10, where “zero” represented no pain and “10” represented worst pain imaginable.

**Figure 3:**
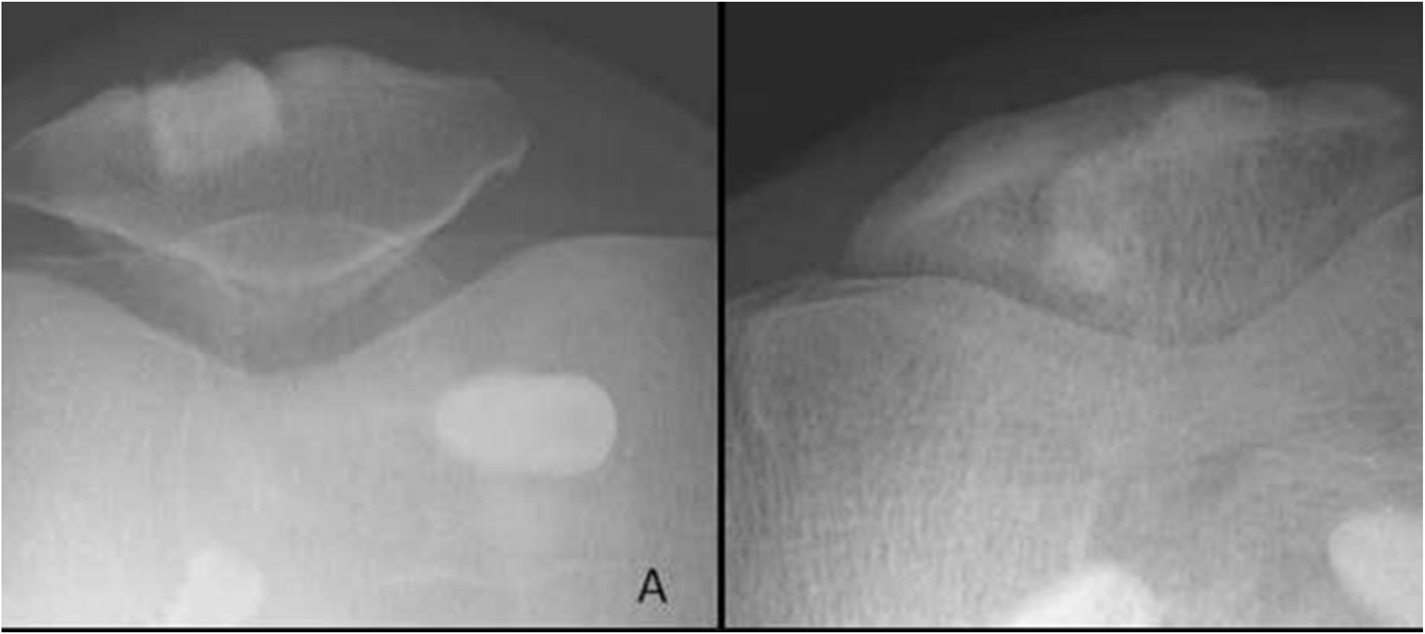
Radiograph showing (A) HAP:BG block in patellar defect of study group after six weeks of surgery, (B) two years later it has completely incorporated into the host bone with normal contour of bone restored.

Radiological evaluation was done using anteroposterior view, lateral view, and skyline view of the knee joint. These radiographs were assessed for HAP:BG block incorporation, dissolution, fragmentation, and migration (Figure 3). The block incorporation was taken as “complete”, if there was establishment of trabeculae across the block, loss of radio-opacity and gradual blurring of the soft margin of the block.

### Statistical analysis

Statistical analysis was performed using SPSS (SPSS Inc, Chicago, Illinois). The Chi-square and Independent *T*-test were used to compare different parameters in both groups. *P*-value of less than 0.05 was considered significant.

## Results

Total 31 patients were recruited in this study, with 15 patients in the study group and 16 patients in the control group. All patients in both groups were male and the mode of injury was sports activity. The mean age of patients in control group was 32.4 years (20–43 years) and in the study group it was 27.8 years (19–43) (*p* < 0.05). All patients included in this study were available for follow-up.

On clinical evaluation at two years, no statistical significance was noted between the IKDC scores of each group for subjective knee evaluation (Table 2). The final grade in IKDC evaluation (Table 3) did not show a notable difference between the groups. Evaluation of specific donor-site morbidity revealed patellar donor-site tenderness in 40% (6 of 15) of patients in the study group and 37.5% (6 of 16) of patients in the control group. Pain on kneeling was present in 33.3% (5 of 15) of patients in the study group and 37.5% (6 of 15) in the control group. The knee walking test showed that 40% (6 of 15) in the study group and 43.8% (7 of 16) in the control group had pain. There was no statistically significant difference between the two groups in terms of patellar donor-site tenderness, knee pain, and knee walking test with *p* > 0.05 (Table 2). The mean visual analogue score for knee pain was 3.0 in the study group and 3.13 in the control group, with no statistically significant difference (*p* > 0.05) (Table 2). Retropatellar pain was present in 20% (3 of 15) patients of the study group and 25% (4 of 16) of the control group.

**Table 2:** Donor-site assessment and IKDC Subjective knee evaluation scores at two years follow up.

	Study group 1	Control group	*p* Value
Local Tenderness	NT – 9 (60%)	NT – 10 (62.5%)	0.589
	T – 6 (40%)	T – 6 (37.5%)	
Kneel pain	NP – 10 (66.7%)	NP – 10 (62.5%)	0.553
	P – 5 (33.3%)	P – 6 (37.5%)	
Knee walking pain	NP – 9 (60%)	NP – 9 (56.3%)	0.561
	P – 6 (40%)	P – 7 (43.8%)	
VAS	3.0	3.13	0.677
IKDC Subjective assessment score	71.4	70.9	0.676

T – Tender, NT – Non tender, P – Painful, NP – Not painful, VAS – Visual analogue score.

**Table 3:** Final Grade of IKDC knee evaluations at two years follow up.

IKDC grade	Study group	Control group	*p* Value
A	4 (26.6%)	5 (31.25%)	0.961 (Chi-Square test)
B	9 (60%)	9 (56.25%)	
C	2 (13.3%)	2 (12.25%)	
D	0	0	

**Figure 4:**
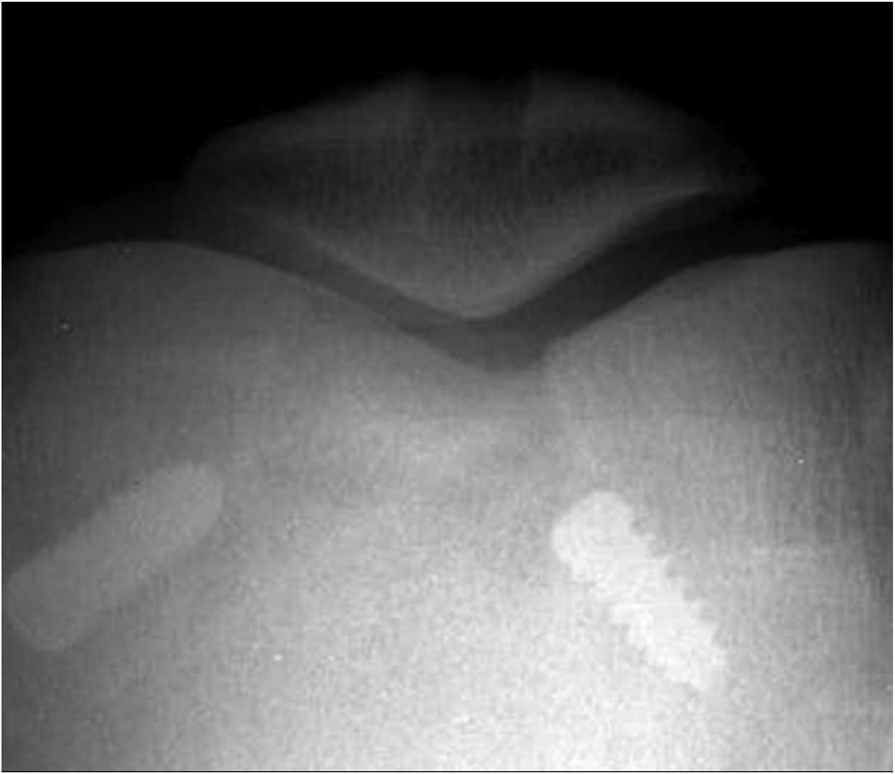
Defect noted in radiograph of control group patient even at two years of follow-up.

The radiological assessment showed that in the study group HAP:BG blocks incorporated into the host bone in all cases reforming the bone to near normal contours (Figure 3). There was no fragmentation or dissolution of the ceramic block (Figure 3). In the control group, the bone defect was visible even at the end of two years (Figure 4). There was no incidence of deep infection, patellar fracture, or patellar-tendon ruptures in any of the patients of both the groups. Complications included superficial wound infection of the tibial graft harvest site in one patient of the study group, which healed with debridement and antibiotics. In the control group, one patient had coarse patellofemoral crepitus on clinical examination, but was asymptomatic. The patient of the control group who had bony spur in the lower pole of patella on radiograph was asymptomatic, and it was not tender on palpation. Only one patient in each group had more than 3° loss of extension.

## Discussion

The most common problem following BPTB graft harvest for ACL reconstruction is anterior knee pain [5–8] and causes morbidity for patients who require kneeling for occupation and religious purposes [7, 14]. Anterior knee pain has been correlated with loss of motion, loss of extensor mechanism, and patient’s dissatisfaction [15–18].

In an attempt to reduce the symptoms, researchers used two incision techniques, contralateral side graft harvest and refilling the defect with cored cancellous bone graft [15, 19]. The effects of bone graft substitutes on the donor-site morbidity have not yet been evaluated. In one study where Osteoset^®^ (Wright Medical) was used as a bone graft substitute for refilling donor-site defects of BPTB graft harvest site, it was not found to be effective in new bone formation [20]. However, this study only evaluated the bone formation in the defect and did not assess the donor site morbidity or subjective functional outcome [20]. On evaluation of two groups of patients in this study, where the bony defects of the donor sites were filled up with HAP:BG in the study group and without filling in the control group, no significant differences in terms of anterior knee pain, tenderness, and knee walking test were elicited. Thus, it became quite evident that filling the defect with bone graft substitute does not necessarily reduce the symptoms of donor site morbidity.

Previous reports on use of bone graft for filling the patellar defect of donor site was also not encouraging. In a prospective randomized study, Brandsson et al. have shown that suturing the patellar tendon defect and bone grafting the defect in the patella did not reduce anterior knee problems or donor-site morbidity [21]. Boszotta and Prunner also found that bone grafting the patellar defect did not reduce kneeling complaints or patello-femoral problems [17].

We presume that some other factors are responsible for the anterior knee pain and tenderness following BPTB graft harvest. Quadriceps’ weakness and flexion contracture have been reported after BPTB graft fixation use in ACL reconstruction, and researchers believe that this is because of the alteration of the biomechanics of the patella-femoral joint following the graft harvest [18]. Irrgang and Harner stated that loss of extension contributes to anterior knee pain [26]. In our study only one patient in each group had more than 3° loss of extension but still about 20% in the study group and 25% in the control group had anterior knee discomfort. Thus, the anterior knee symptoms and patello-femoral problems could have multifactorial genesis as proposed by Ritchie and Parker [22]. Injury to the infrapatellar branch of saphenous nerve has been implicated as one of the important causes for the anterior knee discomfort following BPTB graft harvest [7, 15]. Researchers have reported injury of this nerve and the nerve branches to the periosteum causing neuroma formation with midline incision, used for BPTB graft harvest. The standard midline incision in this study remained uniform for both the groups of patients and we believe that this could be one of the factors responsible for anterior knee symptoms. Bone grafting the patellar donor-site defect has been reported to prevent late patellar fractures [13]. The role of bone graft substitute in prevention of patellar fracture is difficult to interpret from the present study. None of the patients in both groups had any patellar fracture. We believe that the technique of BPTB graft harvest is more important rather than filling the defect in prevention of patellar fracture [23–25]. An oscillating saw was used for the removal of the bone blocks in this study. This technique leaves a bone defect on the removal site which is characterized by two edges on the tibial tuberosity and patella; these are palpable through the skin if not refilled. Refilling the defect with ceramic block has significantly improved the cosmetic appearance as the bone defects had been completely filled. Refilling the defect with bone graft has shown variable results. Kohn and Sander–Beuermann found signs of spontaneous closure of the central gap in the patellar tendon up to 2.5 years after the operation [8]. Using ultrasonography these authors noted a normal patellar tendon anatomy two years after closure of the paratenon and bone grafting of the patellar defect [8]. However, painful bone spurs developed at the apex of the patella in some of their patients. Contrary to it, Boszotta and Prunner reported improved cosmetic appearance after refilling the donor defect with autograft and did not find any depression or bone spurs [17]. We closed the paratenon in both groups of our patients and other than filling the defect with bone graft substitute in the study group, no surgical technique differences were there between the patients. We found depression defect in all patients of the control group, but no such depression or irregularities like bony spur were seen in the study group. One patient in the control group had a prominent bony spur as well. It is important to shape the HAP:BG blocks according to the defect with the help of bone rasp to prevent them from extending beyond the lower pole of patella and this can ensure a perfect fit in the defect. These blocks should be in firm contact with the recipient bone to allow early and complete incorporation. The radiographs showed incorporation of the HAP:BG bone graft substitutes in all our patients. We also observed that HAP:BG bone graft substitute does not compromise the functional outcome. The IKDC scores in our study showed satisfactory recovery and subjective assessment in both groups.

There are few limitations in this study. The number of patients recruited in this study is small. Besides that, randomization of the patients could have improved the quality of the study. The control group patients were older compared to the study group patients. However, all the patients were young adults and below 43 years of age. Despite these limitations, this prospective preliminary report has reached to a conclusion which is statistically significant. Evaluation by a blinded investigator with a control group is the strength of this report.

This preliminary study showed that HAP:BG blocks do not reduce the anterior knee pain symptoms when used for filling the bone defects of donor site after ACL reconstruction surgery with autogenous BPTB graft. Other than regular filling of the defect, it has no additional benefits in reducing the donor-site morbidities.

## Conflict of interest

There are no conflicts of interest to declare.
